# Gold as a Filling Material, with Special Reference to Plastic Gold

**Published:** 1909-05-15

**Authors:** C. G. Morsheimer

**Affiliations:** Rochester, N. Y.


### GOLD AS A FILLING MATERIAL, WITH SPECIAL REFERENCE TO PLASTIC GOLD.

BY C. G. MORSHEIMER, D.D.S., ROCHESTER, N. Y.

Read before Rochester Dental Society.

Gold, At. Wt. 195.7. fusing pt. 1199. Centegrade. Gold is one of the few metals found in the metallic state, and probably one of the first known to men. Allusions to it are frequent in the old Testament, and jewelry and vessels found in Egyptian tombs afford evidence of the perfection attained in working it at a period ealier than the government of Joseph. There are many evidences that processes of alloying, refining and separating gold were practiced at a very early period of the worlds history. According to Pliny, the metalurgy of gold was known in his day. Vitruvius also gives a detailed account of the method of recovering gold by amalgamation from cloth into which it had been woven. It was employed in Rome for the purpose of fixing artificial teeth more than three hundred years before the Christian era, and a law of the ''twelve tables'' makes exception with regard to such gold, permitting it to be buried with the dead. The great beauty of color and lustre and the power of resisting oxidation which gold possesses have caused it to be valued from the earliest ages for the purpose of adornment and as a circulating medium.

There are several kinds of gold used for the filling of the teeth each one having its place where different conditions exist. Non-cohesive or soft gold should be used in cavities having four walls with a flat base. In preparing these cavities I use a round or rcse bur at first and shape the cavity with the inverted cone bur. This kind of gold can be manipulated much faster than Cohesive gold, as larger pieces may be used. It seals the cavity tighter and can be burnished easier to the walls. Union does not take place between the particles of gold introduced into the cavity; they are simply made to adhere mechanically by wedging the mats against each other into the cavity properly prepared to retain them

Cohesive gold is emplcyed largely for building up contour and approximal fillings. With this gold the particles cohere and the filling is a homogenious mass; each piece is welded upon the other with the hand or automatic mallet; the cavity is prepared with grooves and undercuts for starting and retaining the filling.

Another method of filling the teeth with gold is by means of the cast inlay made of pure or 22K. gold; it is an ideal one, if in the hands of a competent operator. The cavity should be prepared so that the wax inlay can be removed without changing its shape, and still be self retentive. The advantages are that it can be done without the use cf the rubber dam, and long tedious operations of hours at a sitting are eliminated. The frail walls of the cavity are cut back so the stress is brought more direct up on the gold, thereby protecting the cavity walls. This method I have used over one year without any failure that I know of.

The crystal gold, including ''Watts'' is cohesive and must be treated as such. It is prepared by passing an electric current of feeble intensity through a solution of chloride of gold (which is pure gold dissolved in Aqua Regia) in which a plate of pure gold forming the anode is suspended and a platinum plate forming the cathode. The solution is decomposed and the gold is deposited on the platinum plate in the form of crystals, which vary in size according to the strength of the solution and intensity of the current. As the solution loses gold by deposition of the metal, it is replenished from the suspended gold plate, which is gradually dissolved. The crystals thus formed are generally pure; they are collected, washed and dried, and the product is ready for use. A crystallized form of gold is also obtained when an amalgam of gold is slowly heated until the whole of the mercury is expelled. When, however, a light spongy mass is required, the amalgam is first treated with nitric acid to dissolve out the excess of mercury. The crystals thus obtained are then heated to expell the remaining mercury, a method that leaves the gold ''spongy'' as mentioned and with a bright lusterous appearance. As I understand it, ''Moss Fibre'' gold is prepared by this method.

In the making of Sponge gold, metallic gold may be precipitated from a solution of gold chloride in the form of fine powder, in scales, in a more or less crystallized state, or in a spongy condition, according to the nature of the precipitating agent employed the strength of the solution, and mode to be pursued in manipulating it. When sulphurous acid is used as a precipitant, the gold is in the form of a brown powder, which is scaly. When oxalic acid is used the precipite is of several forms. Ferrous sulphate is similarly used. The ''Plastic Golds'' most used are ''Keton Williams,'' ''Watts' Crystal'' and ''Moss Fibre.''

Keton Williams gold should be thoroughly annealed on a mica tray. The filling is very easy to start with this gold, and can be made in much less time than with any other. I always fill the base and larger portion of the cavity with it and finish with Watts' crystal, which requires but little annealing and gives a much better finish. These golds that are broken up chemically are divided into smaller particles than the foil gold, therefore I think one can get a better condensed and a more homogenous filling.

